# WP1066 Sensitizes Oral Squamous Cell Carcinoma Cells to Cisplatin by Targeting STAT3/miR-21 axis

**DOI:** 10.1038/srep07461

**Published:** 2014-12-17

**Authors:** Xuan Zhou, Yu Ren, Aiqin Liu, Rui Jin, Qingping Jiang, Yuanyuan Huang, Lingping Kong, Xudong Wang, Lun Zhang

**Affiliations:** 1 The Department of Otorhinolaryngology and Maxillofacial Oncology; Tianjin Medical University Cancer Institute & Hospital, Key Laboratory of Cancer Prevention and Therapy, Tianjin Cancer Institute; National Clinical Research Center of Cancer; Tianjin 300060, P.R. China; 2 Tianjin Research Center of Basic Medical Science, Tianjin Medical University, Tianjin 300070, P.R. China; 3 Department of Pathology, the third affiliated hospital, Guangzhou medical university, Guangzhou 510150, P.R. China

## Abstract

Accumulating evidence reveals that activation of STAT3 and miR-21 contributes to chemoresistance in multiple tumors. We examined the expression of STAT3 and miR-21 in 43 oral squamous cell carcinoma (OSCC) tumors and classified them into cisplatin sensitive or resistant group. Tca8113 and Tca8113/DDP cells were treated with cisplatin (DDP), WP1066 (STAT3 inhibitor) or in combination. MTT, colony formation, wound healing, 3-D culture, and transwell chamber assays were used to evaluate the malignant phenotype of OSCC cells. We evaluated the effect of WP1066 on the expression of STAT3 and miR-21. A Tca8113/DDP OSCC xenograft tumor model was established to evaluate the therapeutic effect of WP1066 in combination with DDP. The expression of STAT3/miR-21 was significantly increased in DDP-resistant OSCC samples and Tca8113/DDP cells compared to its parental cell. Treatment of DDP combined with WP1066 efficiently inhibited Tca8113 and Tca8113/DDP cell proliferation, migration and invasion. STAT3 mediated OSCC cell survival and DDP resistance through upregulating the expression of miR-21 and downregulating miR-21 downstream targets, including PTEN, TIMP3 and PDCD4. WP1066 plus DDP treatment could inhibit Tca8113 and Tca8113/DDP cell growth by inhibiting STAT3 phosphorylation and miR-21 expression. These results indicated that STAT3/miR-21 axis could be a candidate therapeutic target for OSCC chemoresistance.

Oral squamous cell carcinoma (OSCC) is the most common type of head and neck cancer^1^. The 5-year survival rate of the oral tongue cancer is about 53%^2^. Cis-Dichlorodiamineplatinum (DDP) is the first-line choice for head and neck squamous cell carcinoma (HNSCC) including OSCC. However, 70 to 80% of patients with relapsed or recurrent disease present resistant to DDP^3^,^4^. Many oral cancer patients experience recurrent disease after initial therapy and become refractory to multiple chemotherapeutic drugs. Thus, there is an urgent need to better understand the molecular mechanism underlying chemoresistance.

The cellular sensitivity to DDP is influenced by a number of factors, including genes related to apoptosis and DNA damage-repair, chaperones, transporters, cell cycle checkpoints, transcription factors, oncogenes, small GTPases, GSH enzymes, cytoskeletal proteins, and mitochondria components^5^. Among them, STAT3 protein is a cytoplasmic transcription factor that translocates into the nucleus upon cytokine activation, which plays important roles in proliferation, differentiation and apoptosis^6^,^7^. STAT3 has been validated to affect cancer cell sensitivity to DDP^8^, paclitaxel^9^, imatinib^10^, and gefitinib^11^. Our previous data indicates that by suppressing STAT3 activation, HNSCC shows an increased sensitivity to DDP *in vitro*^12^.

MicroRNAs (MiRNAs) are non-coding RNAs, which functions as translational and post-transcriptional regulators of gene expression^13^. Several miRNAs have been shown to sensitize DDP-resistant cell lines to DDP and other drugs by promoting apoptosis. For example, miR-21 was reported to enhance DDP resistance in gastric cancer by activating PTEN/PI3K/Akt pathway^14^. In gastric and lung cancer cell lines, the miRNA cluster miR-200bc/429 was shown to promote apoptosis by targeting B-cell lymphoma 2 (Bcl-2) and X-linked inhibitor of apoptosis protein (XIAP), which sensitized those resistant cell lines to vincristine as well as DDP^15^.

STAT3/miR-21 axis is the most extensively studied STAT3/miRNA interaction. It is well established that miR-21 is induced by IL-6/STAT3 activation in chronic lymphocytic leukemia^16^, glioma^17^, HNSCC^18^ and multiple myeloma^19^. In the present study, we explored whether human OSCC chemo-resistance can be reversed through targeting STAT3/miR-21 axis using WP1066 reagent. WP1066 was first introduced in 2007^20^,^21^ as a JAK kinase inhibitor in acute myelogenous leukemia and glioma, and now it is used as a STAT3 phosphorylation (Tyr 705 residue) blocker. As expected, the subsequent results demonstrate that STAT/miR-21 pathway can be treated as a candidate target for chemo-sensitivity in OSCC.

## Results

### STAT3 and miR-21 was over-expressed in DDP resistant OSCC tumor tissues

**Figure 1:**
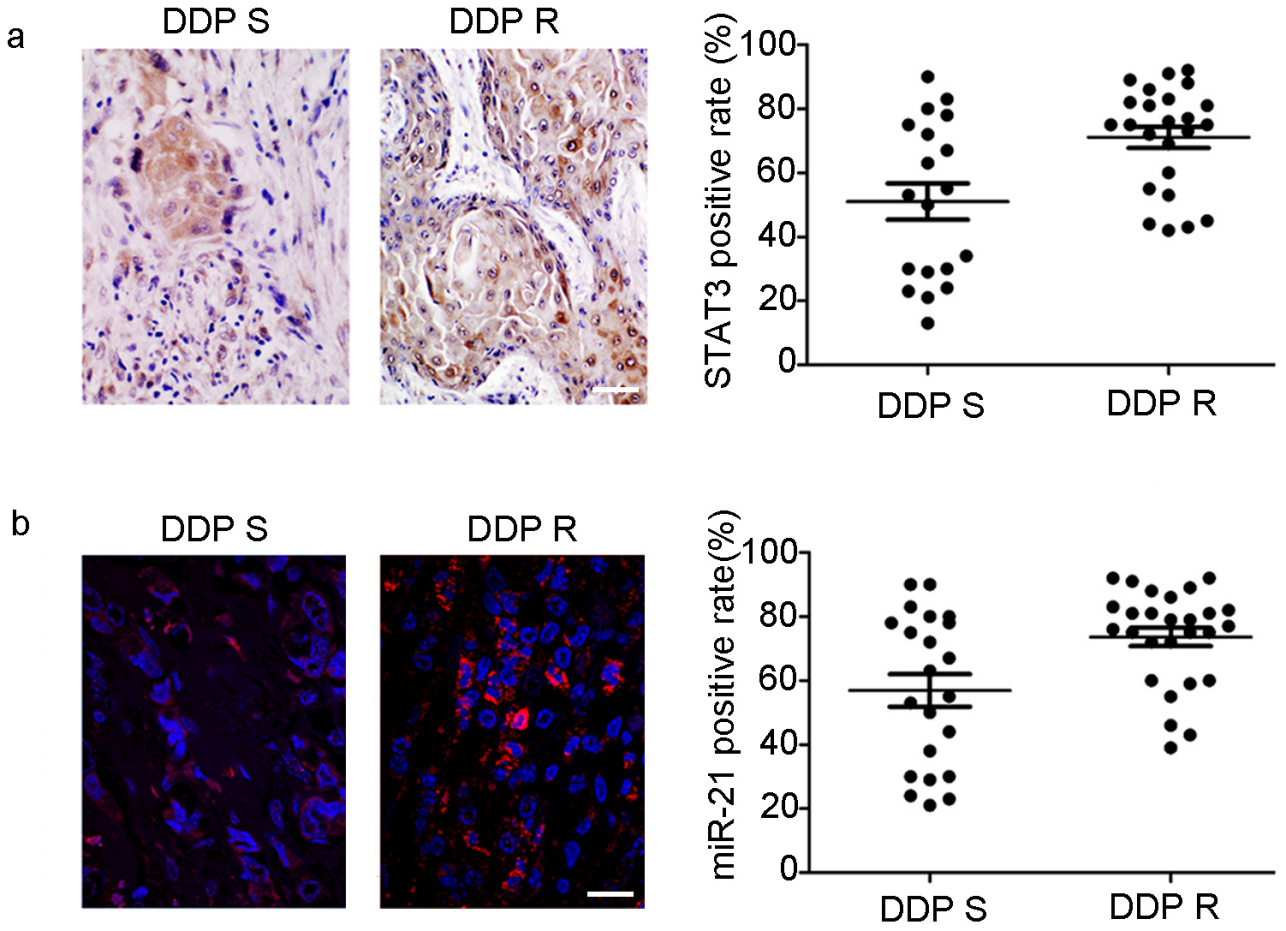
STAT3 and miR-21 is over-expressed in DDP resistant OSCC tumor tissues. (a) In DDP resistant OSCC tumor tissues, STAT3 was highly expressed in the cytoplasm and nucleus of tumor cells than in DDP sensitive OSCC tissues (*P* < 0.05). (B) In DDP resistant OSCC tissues, ISH staining reveals that miR-21 (red) has a relatively high expression than in DDP sensitive OSCC tissues (*P* < 0.05) (Bar = 100 μm).

History of adjuvant chemotherapy indicated that the specimens of 43 OSCC patients displayed diverse sensitivity to DDP. The therapeutic effect was evaluated according to American Joint of Cancer Committee “lip and oral cancer” TNM staging system (2010 version) and RECIST (Response Evaluation Criteria in Solid Tumors, http://chemoth.com/recist). There were 19 patients sensitive to DDP and 24 resistant. Within the sensitive group, 5 of 19 patients (26.32%) had high STAT3 expression, whereas in the DDP-resistant group, 17 of 24 cases (70.83%) showed high STAT3 expression. A significant difference in expression level of STAT3 between the DDP-sensitive and -resistant group was observed (*P* < 0.05; Figure 1a).

Furthermore, we measured the expression level of miR-21 in the same specimens of the 43 OSCC patients using an ISH assay. Only 7 of 19 patients (36.8%) had high miR-21 expression in the DDP sensitive group, whereas 21 of 24 cases (87.5%) had strong miR-21 expression in the DDP-resistant group. A significant difference in the expression level of miR-21 between the DDP-sensitive and -resistant group was detected (*P* < 0.05; Figure 1b).

### STAT3/miR-21 axis was upregulated in the DDP-resistant Tca8113/DDP

**Figure 2:**
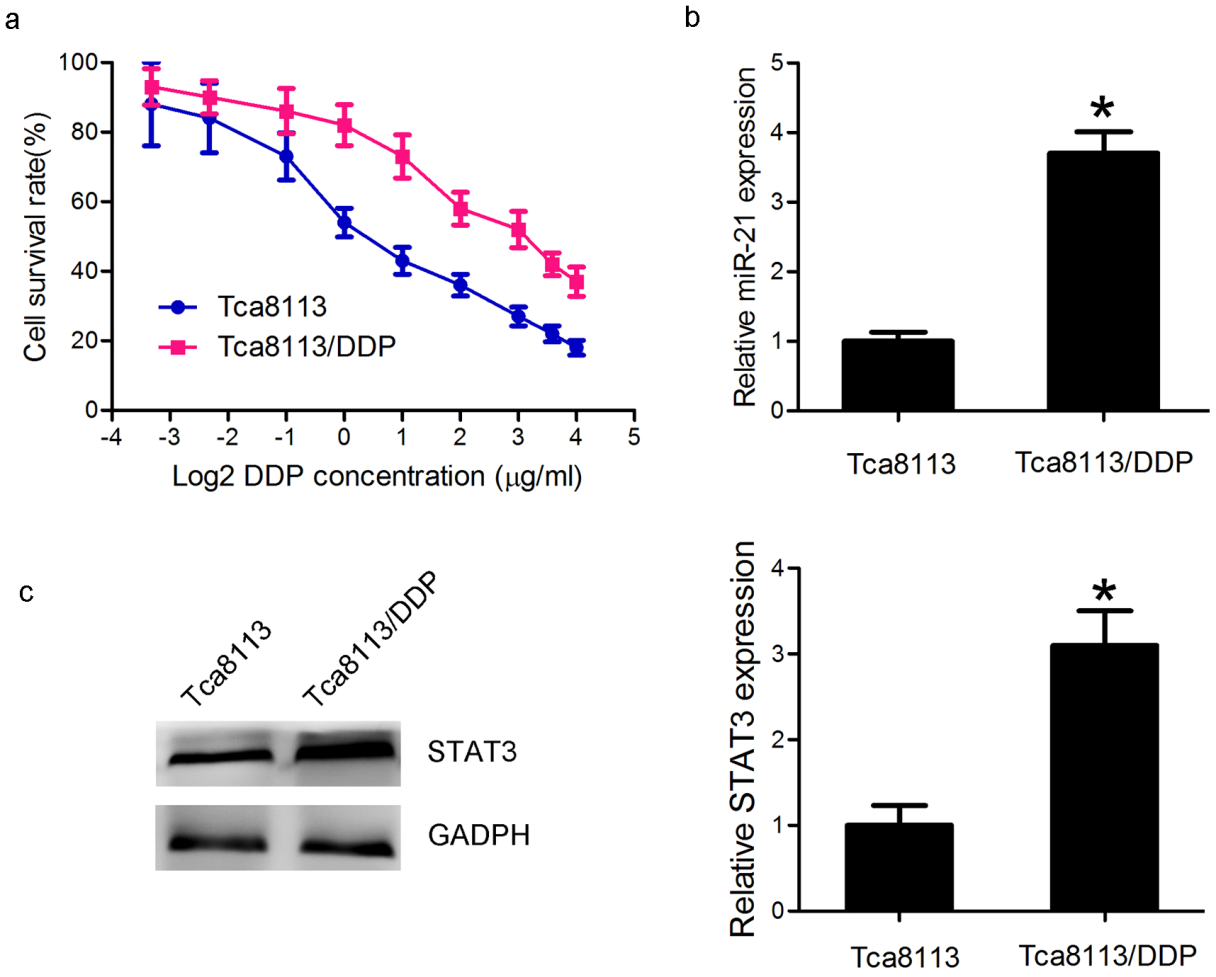
STAT3/miR-21 axis was upregulated in the DDP-resistant Tca8113/DDP cells. (a) MTT assay indicated that Tca8113/DDP and Tca8113 cells showed different sensitivity to DDP treatment. The cells were incubated in different doses of DDP (0.1, 0.2, 0.5, 1, 2, 4, 8, 12 and 16 μg/ml) for 48 h, and survival rate was determined by MTT assay. (b) QPCR showed that miR-21 was 3.7 folds higher in Tca8113/DDP cells than in Tca8113 cells (*P* < 0.05). (c) Western blot assay indicated that STAT3 protein expression was 3 folds higher in Tca8113/DDP cells than in Tca8113 cells (*P* < 0.05). Anti-GAPDH antibody was used as a loading control.

The survival curves of the Tca8113/DDP and the parental Tca8113 cell lines were shown in Fig. 2a. The Tca8113/DDP cell lines showed 10.67-fold increased acquired resistance to DDP based on IC50 (9.6 μg/ml vs. 0.9. μg/ml, *P* < 0.05). To investigate the involvement of miR-21 in DDP resistant, we conducted qPCR analysis to examine the expression level of miR-21. We found that the miR-21 expression level was 3.7 folds higher in the Tca8113/DDP cells than in the Tca8113 cells (Fig. 2b, *P* < 0.05), consistent with the previous report^14^. In addition, Western blot showed that STAT3 expression level in Tca8113/DDP cells was approximately 3 folds higher than in Tca8113 cell (Fig. 2c, *P* < 0.05). Based on these results, we hypothesized that miR-21 and STAT3 could be associated with DDP resistance in OSCC cells.

### WP1066 potentiated DDP efficacy in DDP resistant OSCC cell line *in vitro*

**Figure 3:**
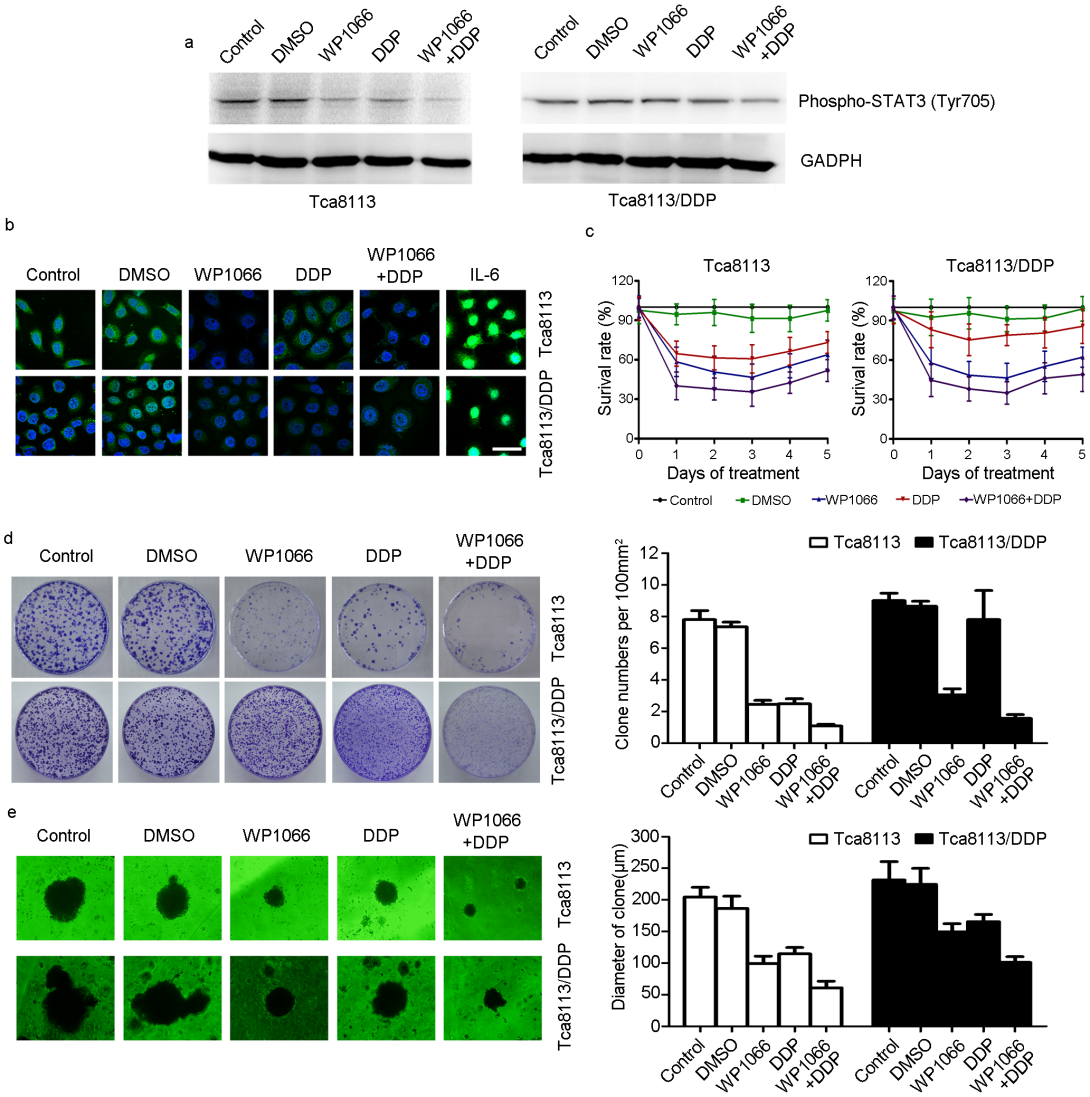
WP1066 sensitized the Tca8113/DDP cells to DDP. (a) Tca8113 and Tca8113/DDP cells were treated with 5 μM WP1066 and harvested at 48 h. Western blots showed WP1066 and DDP combined treatment inhibited p-STAT3 expression in both cell lines. Anti-GAPDH antibody was used as a loading control. (b) STAT3 was measured by immunofluorescence (IF) method. In WP1066 alone and DDP+ WP1066 treated group, STAT3 expression was downregulated significantly in both Tca8113 and Tca8113/DDP cells. In IL-6 treated cell, STAT3 expression was upregulated (Bar = 20 μm). (c) MTT assay indicated that WP1066 and DDP combined treatment inhibited both Tca8113 and Tca8113/DDP cell lines growth *in vitro*. (d) Clone formation assay indicated that Tca8113/DDP cells were resistant to DDP. DDP + WP1066 treatment inhibited Tca8113/DDP and Tca8113 cell growth effectively. (e) 3-D culture assay showed that DDP + WP1066 treatment significantly inhibited Tca8113/DDP and Tca8113 cell growth on martrigel matrix.

**Figure 4:**
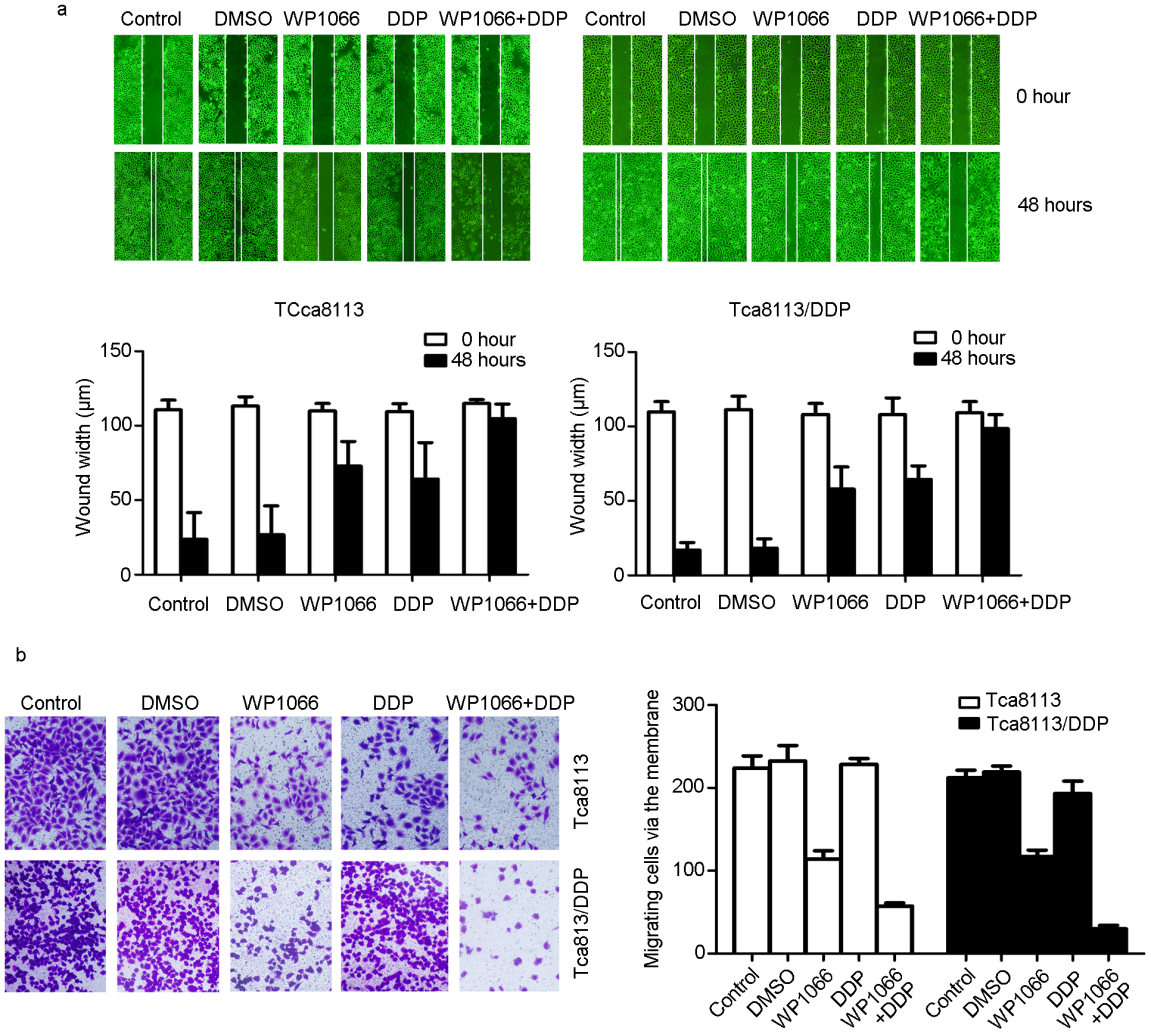
WP1066 sensitized OSCC cells to DDP and reduced migration capability. Scratch (Wound healing) and transwell assays were used to assess cell migration and invasion capability at 48 h after treatment. (a) Scratch assay has shown that combination of WP1066 and DDP could delay the scratch healing in Tca8113 and Tca8113/DDP cells. (b) Transwell assay has shown that combination of WP1066 and DDP could decrease the invasion of Tca8113 and Tca8113/DDP cells.

To evaluate the combined effect of DDP with STAT3 inhibitor, Tca8113/DDP cells were treated with DDP alone, WP1066 alone or a combination of DDP and WP1066. In Tca8113/DDP cells, combined treatment of DDP with WP1066 was more effective compared to either DDP or WP1066 treatment alone on inhibiting phospho-STAT3 expression (Figure 3a). Additionally, IF staining indicated that STAT3 expression was inhibited more effectively by combined therapy than single treatment. And STAT3 nuclear accumulation could be stimulated by IL-6 treatment (Figure 3b). MTT assay indicated in both cell lines, combination of DDP and WP1066 treatment inhibited cell proliferation ability (Figure 3c). The clone formation assay showed that Tca8113/DDP cells treated with DDP and WP1066 in combination had a significantly lower survival rate (Figure 3d, *P* < 0.05). The 3-D martrigel culture assay showed that the diameters of cell clones of DDP + WP1066 treated cells were significantly reduced than other groups (Figure 3e, *P* < 0.05). Scratch assay (Figure 4a, *P* < 0.05) and transwell chamber assay (Figure 4b, *P* < 0.05) showed similar results, demonstrating that the combination of WP1066 and DDP could inhibit Tca8113/DDP cell migration and invasion.

**Figure 5:**
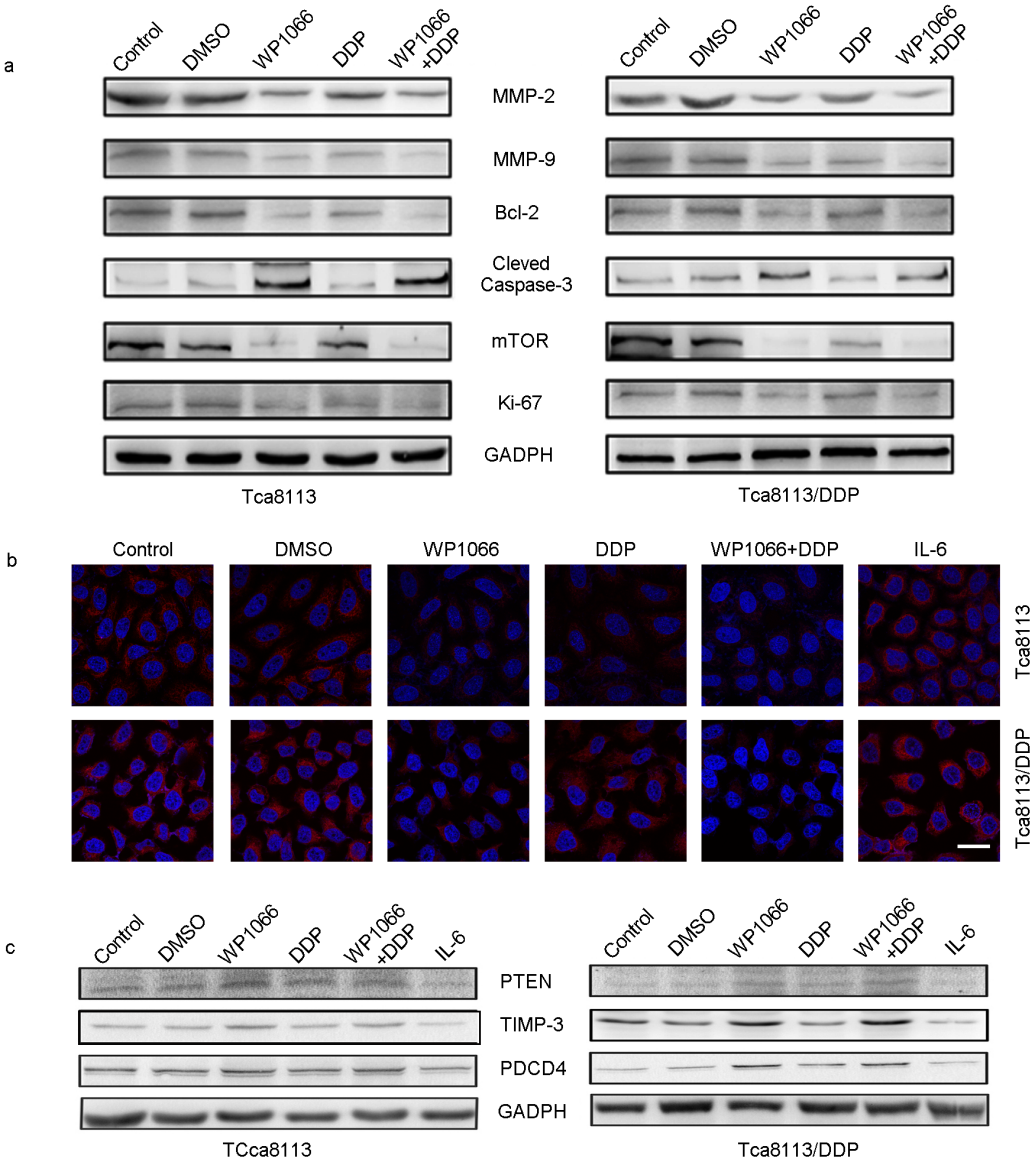
WP1066 combined DDP could inhibit the expression of miR-21 and its target genes. ISH was used to determine the expression of miR-21 in treated OSCC cells; and Western blot was used to detect the expression of its target genes. (a) In Tca8113 and Tca8113/DDP cells, WP1066 combined with DDP treatment could inhibit MMP2/9, Bcl-2, mTOR, Ki67 expression, while in DDP + WP1066 treated group, Caspase-3 expression was upregulated. (b) In Tca8113 and Tca8113/DDP cells, WP1066 combined with DDP treatment could induce the expression of miR-21 (Red signal labeled by Cy3, cell nuclei was labeled by DAPI). IL-6 treatment could inhibit the expression of miR-21. (Bar = 20 μm) (c) In Tca8113 and Tca8113/DDP cells, DDP + WP1066 treatment could induce the expression of PTEN, TIMP3 and PDCD4. While IL-6 treatment could inhibit their expression in both cell lines. The gels were cropped, but those gels were run under the same experimental condition.

In Tca8113/DDP cells treated with DDP + WP1066, protein expression levels of Ki-67, MMP2/9, Bcl-2, and mTOR were significantly downregulated while the caspase-3 level was upregulated (Figure 5a). DDP combined with 5 μM of WP1066 exhibited a strong synergistic effect, which reduced the protein levels of pSTAT3 (Figure 3a) and miR-21 (Figure 5b). These results suggested that WP1066 reversed DDP resistance in Tca8113/DDP cells by inhibiting the activation of STAT3/miR-21 axis. To confirm the involvement of miR-21 in STAT3 mediated DDP resistance, we examined the expression levels of its target genes including PTEN, TIMP3 and PDCD4. We found that PTEN, TIMP3 and PDCD4 protein were upregulated in DDP + WP1066 treated groups (Figure 5c). Also, IL-6 induced activation of STAT3 triggered miR-21 expression in Tca8113/DDP and Tca8113 cells (Figure 5b).

### Combination of WP1066 and DDP inhibits Tca8113/DDP xenograft tumor growth

**Figure 6:**
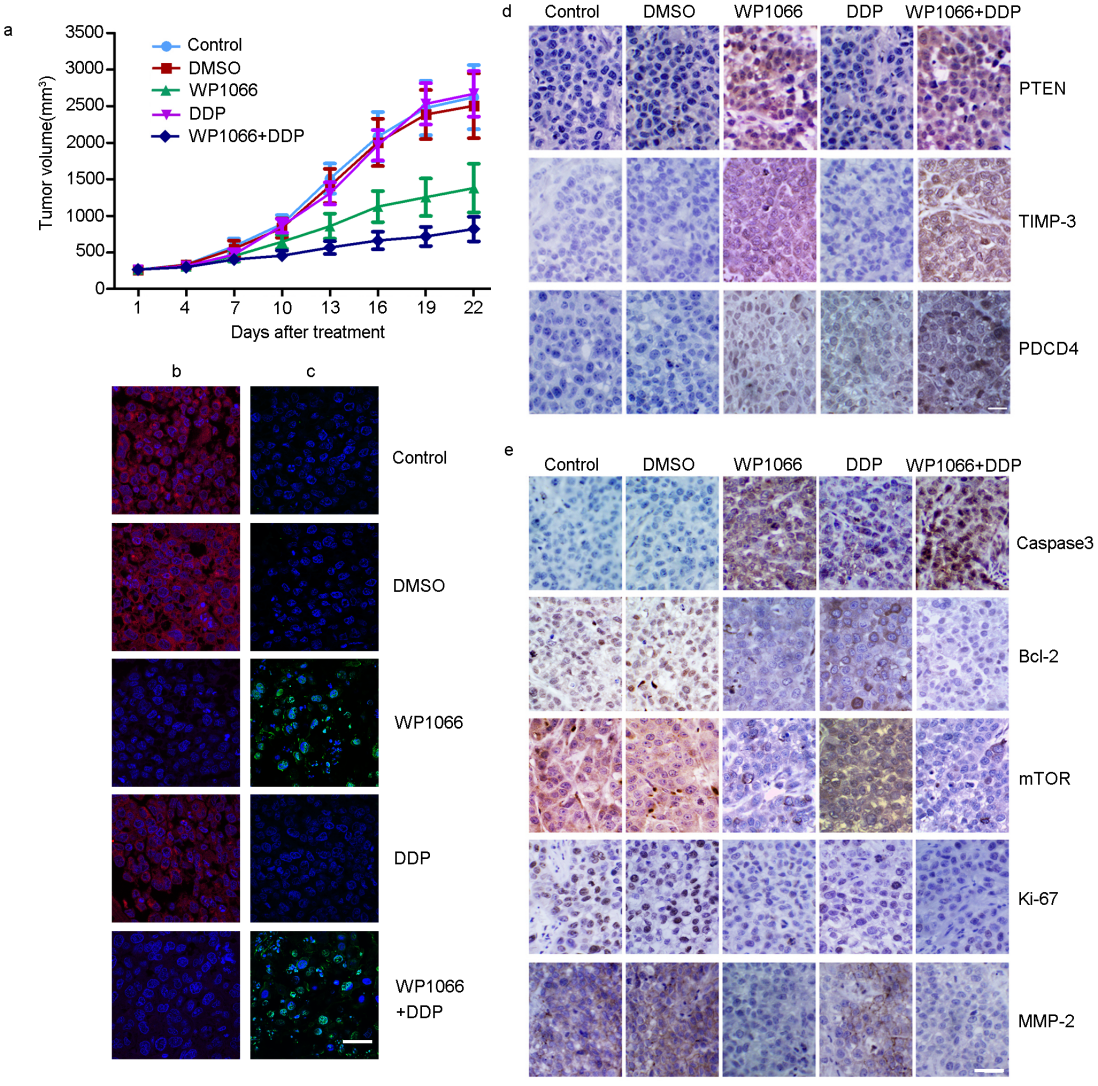
Combination of WP1066 and DDP inhibited Tca8113/DDP xenograft tumor growth. (a) The growth curves of blank control, DMSO, WP1066-treated, DDP, and DDP + WP1066 treated Tca8113/DDP xenograft tumors. The growth rate in WP1066, DDP and DDP + WP1066 group were inhibited compared to control and DMSO groups. (b) ISH showed that, in WP1006, WP1066 and DDP treated Tca8113/DDP tumors, miR-21 expression was inhibited (Red signal labeled by Cy3, cell nuclei was labeled by DAPI). (c) TUNEL assay showed an induced apoptotic nucleus (green) in WP1066 combined DDP-treated Tca8113/DDP tumors. (d) IHC staining showed the upregulation of PTEN, TIMP-3, and PDCD4 in WP1066-treated and DDP + WP1066 treated group.(e) IHC assay was used to determine the expression of tumor growth related proteins in Tca8113/DDP xenograft tumors. Downregulation of mTOR, Ki67, Bcl-2, and MMP-2 was observed in WP1066 and WP1066 combined DDP-treated group. In combined treated group Caspase-3 expression was upregulated. (Bar = 100 μm).

We employed a Tca8113/DDP xenograft tumor model to confirm the therapeutic potential of WP1066 in combination with DDP *in vivo*. Tumor growth curve demonstrated that DDP + WP1066 treatment was effective compared to either DDP or WP1066 alone or DMSO (Figure 6a). IHC staining showed that, in combined treatment group, mTOR, Ki67, Bcl-2, and MMP-2 (Figure 6e) were downregulated whereas PDCD4, TIMP3, PTEN (Figure 6d) and Caspase-3 (Figure 6e) were upregulated. In addition, TUNEL assay illustrated that combination treatment resulted in increased apoptosis (Figure 6c). These results indicated that WP1066 could partially increase the sensitivity of Tca8113/DDP cells to DDP.

## Discussion

In this study, we found that abnormal over-expression of STAT3/miR-21 in OSCC was associated with cellular resistance to DDP. WP1066, an effective small molecular inhibitor of STAT3 phosphorylation, was shown to reverse chemoresistance and increase the sensitivity to DDP in DDP-resistant OSCC cell line.

STAT3 is over-expressed and constitutively activated in many types of malignancies, such as breast cancer, HNSCC and glioma. STAT3 plays an important role in oncogenesis by up-regulating the transcription level of molecules promoting tumor cell survival, anti- apoptosis, cell cycle progression and angiogenesis. Target genes of STAT3 include Bcl-2/Bcl-XL^22^,^23^, c-myc^24^, Mcl-1^25^, cyclin D1^26^, vascular endothelial growth factor (VEGF)^27^ and human telomerase reverse transcriptase (hTERT)^28^. Our study demonstrated a high STAT3 expression in 17 of 24 DDP-resistant OSCC specimens. WP1066 + DDP treated Tca8113 and Tca8113/DDP cell lines displayed significant reduction in cell proliferation, migration and invasion compared to those cell lines treated with either DDP or WP1066 alone. More important, combined therapy could increase cellular sensitivity to DDP, through blocking the STAT3 signaling pathway. A similar study has reported that a STAT3 small molecular inhibitor can reverse the DDP-chemoresistance in ovarian cancer^29^.

Recently several miRNAs (miR-21, miR-155, and miR-181b) have been reported to be involved in STAT3-mediated tumorigenesis^30^. Through binding to STAT3-binding sites within the promoters of these oncomiRs, STAT3 activates their transcription and mediates tumorigenesis. MiR-21 was reported to play an important role in chemoresistance to DDP^14^, paclitaxel^31^, and 5-fluorouracil^32^. Multiple genes, for example PTEN, PDCD4, TMP1 and TIMP-3, have been validated as targets of miR-21. Our data showed that miR-21 was overexpressed in DDP-resistant OSCC patients, which was associated with STAT3 overexpression. In WP1066 treated tumors, miR-21 was downregulated, whereas its downstream target genes were upregulated. Moreover, PTEN was a key regulator to PI3K/AKT/mTOR pathway, which modulates cell survival, differentiation, or chemosensitivity in head and neck cancer^33^. These data suggested that miR-21 inhibition by WP1066 contributed to attenuation of DDP-resistant character in Tca8113/DDP cells both *in vitro* and *in vivo*.

Our findings demonstrated the importance of STAT3/miR-21 axis in mediating the chemoresistance to DDP. A small molecular inhibitor of STAT3 sensitizes both DDP-resistant and parental OSCC cell lines to DDP. This treatment strategy could reduce the dose of DDP usage, and DDP-related undesirable adverse effects, such as hematopoietic function inhibition, gastrointestinal reaction, and hair loss. Taken together, STAT3/miR-21 axis may provide a new drug target to OSCC treatment.

## Methods

### In situ hybridization (ISH) and immunohistochemistry (IHC) staining on tissue samples from patients with OSCC

A total of 43 OSCC radical resection tumor samples were randomly collected at Tianjin Medical University Cancer Institute & Hospital between 2011 and 2012. All the procedures were approved by Institutional review board of Tianjin Medical University Medical Principle Committee. All of the cases were classified into DDP sensitive or resistant group according to the clinical treatment record. Briefly, none of the OSCC patients had received any treatment upon admission. The therapeutic resistance was evaluated after 2 cycles of DDP (40 mg/m^2^) standard regimens which were given at the 1st and 4th week. Tumor volume that changed less than 30% was defined as DDP-resistant tumor, while the tumor that shrank greater than 20% was defined as DDP-sensitive tumor (RECIST; http://chemoth.com/recist).

Paraffin-embedded tumor tissue samples were selected for ISH with Cy3-labeled miR-21 probe (Boster, China) and IHC for STAT3 (Cell signaling, USA) expression. The *ISH* and IHC assays were performed as previously described [20].

### Cell culture and reagents

The human Tca8113 OSCC cell line was purchased from the China Center for Type Culture Collection (Wuhan, China). Tca8113/DDP cell line was kindly provided by Dr. Zhenkun Yu, Beijing Tongren Hospital of Capital Medical University. All cell lines were maintained in Dulbecco's modified Eagle's medium (DMEM) (Invitrogen, USA) supplemented with 10% fetal bovine serum (Gibco, USA). WP1066 (Calbiochem, Germany) and IL-6 (Sigma, USA) were dissolved in DMSO (Solarbo, China) for use and storage. Tca8113 and Tca8113/DDP cells were treated with WP1066 for 48 h at a concentration of 5 μM^34^. IL-6 treated cell line served as a control as described previously^35^.

### qPCR for miR-21

Total RNAs were extracted by using the Trizol Reagent (Life technology, USA) in accordance with the manufacturer's instructions. Expression of mature miR-21 was measured using miR-21 QPCR kit (Genepharm, China). The PCR procedure was performed on the Real-time PCR (Bio-Rad, USA) as described previously^36^. U6 was used as an internal control.

### MTT assay

Tca8113 and Tca8113/DDP cells were seeded into 96-well plates at 4000 cells per well. After drug treatment described above, a total volume of 20 μl MTT (Sigma, USA, 5 mg/mL) was added for 4 h. After removal of the medium and MTT, 200 μl DMSO was added to each well, and the optical density was detected at 570 nm^36^.

### Transwell assay

Cell invasion assays were performed using transwell membranes coated with Matrigel (BD Biosciences). Transfected cells were plated at a density of 3 × 10^4^ cells per well. The lower chamber was filled with 20% FBS. After 24 h, cells remaining in the upper chamber were removed with cotton swabs, while invading cells were fixed with 3% paraformaldehyde (Santa Cruz, USA), stained with crystal violet (Solarbo, China)^37^.

### Scratch wound assay

Tca8113 and Tca8113/DDP cells layers were scratched using a sterile pipette tip to form wound gaps. The wound location in the six-well plates was marked. Cells were photographed to record the wound width (0 h). 48 hours later, photographs will be taken again at the marked wound location to measure the cell migration ability^37^.

### 3-D culture assay

1.5 ml matrigel layer (BD, USA) is dispensed into each well of a 12-well plate. Plate was chilled at room temperature until solid. Then 1.5 ml growth agar layer consisting of 2500 cells was added into each well. Plate was chilled at room temperature again until the growth layer congealed. A further 500 ml culture media containing various concentrations of a-solanine was added on top of the growth layer. Incubate the cells at 37°C and 5% CO2 for 2 weeks and total colonies were counted^37^.

### Wound healing assay

A total of 10,000 cells of each cell line were plated in 6-well plates. When cells grew to confluence, inserts were then removed with sterile forceps to create a wound field of approximately 500 mm. After removing the cellular debris with PBS, cells were exposed to DMSO, DDP, WP1066 and WP1066 + DDP for 48 h. Cell migration were perceived by inverted microscope and photographed. The wound area was scaled by Image Pro Plus 5.0 (Olympus, Japan)^37^.

### Western blot analysis

WP1066 and DDP treated OSCC cells were collected for total protein extraction. Extracted proteins were probed with the primary antibodies against PTEN, PDCD4, TIMP-3, Caspase-3, mTOR (Santa Cruz, USA), MMP-2/9, Bcl-2, Ki67, GADPH (Zhongshan, China), and STAT3/-p (Cell Signal Technology, USA).

### Tca8113/DDP xenograft tumor model and TUNEL assay

All animal experimental protocols were approved by Tianjin Medical University Animal Care and Use Committee. The NU/NU nude mice at age of 4 weeks, female, SPF grade (Vital River Laboratories, China) (n = 50, 10 for each group) were implanted subcutaneously with 5 × 10^6^ Tca8113/DDP cells as described previously^36^. The tumor volume was measured with a caliper every 3 days using a formula (volume = long diameter × short diameter^2^/2). When the volume of xenograft tumors was approximately 250 mm^3^, the tumors is injected with DMSO, DDP (7.5 mg/kg, local injection), WP1066 (40 mg/kg, local injection), and WP1066 combined DDP, every 3 days. After 3 weeks, the mice were sacrificed and the xenograft tumors were removed for formalin fixation and preparation of paraffin-embedded sections.

Apoptosis (programmed cell death) in the tumor specimens of the murine model was examined by TUNEL method using an in situ cell death kit (Roche, USA) according to the manufacture's protocol. Nuclei were counterstained with DAPI reagent (Life technology, USA). Positive cells were visualized using FV-1000 laser scanning confocal biological microscopes (Olympus, Japan).

### Statistical analysis

The result was expressed as mean ± standard error (SE), which represented the average of at least three experiments and each of them was performed in triplicate. Differences between groups were determined by t test. A *P* value less than 0.05 was considered statistically significant.

## Author Contributions

X.Z., X.D.W. and L.Z. planned all the experiments; Y.R. and A.Q.L. performed all the *in vitro* experiments; L.P.K. and Q.P.J. mainly performed the *in vivo* experiments; R.J. collected all the tumor samples; Y.Y.H. performed the computational analysis; X.Z., X.D.W. and L.Z. prepared the manuscript. All authors reviewed the manuscript.
